# Soluble programmed death-1 (sPD-1) and programmed death ligand 1 (sPD-L1) as potential biomarkers for the diagnosis and prognosis of glioma patients

**DOI:** 10.5937/jomb0-24692

**Published:** 2020-10-02

**Authors:** Shujun Liu, Yadi Zhu, Chenxi Zhang, Jiajia Liu, Hong Lv, Guojun Zhang, Xixiong Kang

**Affiliations:** 1 Capital Medical University, Beijing Tiantan Hospital, Laboratory Diagnosis Center, Beijing, China; 2 Beijing Engineering Research Center of Immunological Reagents and Clinical Research, Beijing, China; 3 Beijing Engineering Research Center of Immunological Reagents and Clinical Research, Beijing, China + Beihang University, Lab of Biological Science and Medical Engineering, Beijing, China

**Keywords:** soluble programmed death-1, soluble programmed death ligand 1, glioma, biomarkers, prognosis, prognoza, biomarkeri, gliom, rastvorljivi programirani smrtni ligand 1, rastvorljivi programirani smrtni-1

## Abstract

**Background:**

This study aimed at investigating the feasibility of testing for soluble programmed death-1 (sPD-1) and soluble programmed death ligand 1 (sPD-L1) in serum samples of glioma patients and to evaluate the diagnostic and prognostic value of these two soluble molecules.

**Methods:**

Serum samples collected from 70 glioma patients before surgery were designated as the pre-operative (Pre) group, samples obtained from 90 post-surgery glioblastoma patients were designated as the Post group, and samples from 20 healthy volunteers were used as controls. Peripheral blood sPD-1 and sPD-L1 levels were detected by using ELISA kits and compared among the groups. The associations of these soluble molecule levels with clinicopathological variables and tumour progression were investigated.

**Results:**

Among the three groups, the Pre group had the highest sPD-1 levels, whereas the median sPD-L1 level was significantly lower in the Post group than in the other two groups. The area under the curve (AUC) of sPD-1 (0.762) for diagnosis was similar to that of sPD-L1 (0.718). Higher serum levels of sPD-1 and sPD-L1 were present in samples of patients with more advanced brain tumours. Kaplan-Meier analysis showed that higher serum levels of sPD-1 (>11.14 pg/mL) and sPD-L1 (>63.03 pg/mL) might predict shorter progression-free survival times of glioma patients.

**Conclusions:**

This study showed that sPD-1 and sPD-L1 might be promising predictive biomarkers for the diagnosis and prognosis of glioma patients.

## Abstract (sr)

**Uvod:**

Ovo istraživanje imalo je za cilj da ispita mogućnost testiranja rastvorljivog programiranog smrtnog-1 (sPD-1) i rastvorljivog programiranog smrtnog liganda 1 (sPD-L1) u uzor cima seruma kod pacijenata sa gliomom i da proceni dijagnostičku i prognostičku vrednost ova dva rastvorljiva mole kula.

**Metode:**

Uzorci seruma uzeti od 70 pacijenata sa gliomom pre operacije označeni su kao preoperativna Pre-grupa, a uzorci dobijeni od 90 pacijenata sa glioblastomom posle operacije su označeni kao Post-grupa, dok su uzorci 20 zdravih dobrovoljaca korišćeni kao kontrolna grupa. Nivoi sPD-1 periferne krvi i sPD-L1 su detektovani korišćenjem ELISA kompleta i upoređeni su između grupa. Ispitivana je povezanost ovih nivoa rastvorljivih molekula sa kliničko-patološkim varijablama i napredovanjem tumora.

**Rezultati:**

Od tri grupe, Pre-grupa je imala najviši nivo sPD1, dok je srednji nivo sPD-L1 značajno niži u Post-grupi nego u ostale dve grupe. Područje ispod krive (AUC) sPD1 (0,762) za dijagnozu bilo je slično kao kod sPD-L1 (0,718). Viši serumski nivoi sPD-1 i sPD-L1 su bili prisutni u uzorcima pacijenata sa tumorom mozga u uznapredovalom stadijumu. Kaplan-Majerova analiza je pokazala da viši nivoi sPD-1 u serumu (>11,14 pg/mL) i sPD-L1 (>63,03 pg/mL) mogu ukazati na kraće vreme preživljavanja pacijenata sa gliomom bez progresije.

**Zaključak:**

Ova studija je pokazala da se sPD-1 i sPD-L1 mogu smatrati obećavajućim prediktivnim biomarkerima za dijagnozu i prognozu pacijenata sa gliomom.

## Introduction

Gliomas comprise the majority of malignant brain tumours in adults and are characterized by a high recurrence rate and poor overall survival [1]. According to the World Health Organization (WHO) guidelines, gliomas are categorized into four grades (I to IV), where low-grade gliomas include grade I and grade II, and high-grade gliomas, which are aggressive, include grade III and grade IV glioblastoma (GBM) [2]. Despite the use of comprehensive treatments, including neurosurgical resection, radiotherapy and chemotherapy, the prognosis of glioma patients remains poor, especially GBM, which tends to recur and is associated with short survival time [3].

The discovery of immune checkpoints has provided novel therapeutic targets, which have brought hope, especially for brain tumour immunotherapy [4]. The programmed death-1 (PD-1) protein and its ligand, programmed death ligand 1 (PD-L1), are the most intensely investigated immune checkpoints in the era of immuno-oncology. PD-1 has been reported to be expressed by activated T cells and to mediate immuno-suppression [5]. PD-L1 is overexpressed by tumour cells and interacts with PD-1, which triggers the anergy or apoptosis of T cells and thereby contributes to tumour immune escape and tumour progression [6]. Many clinical studies have demonstrated that the expression of PD-L1 on tumour cells or in the tumour microenvironment is associated with poor clinical prognosis and can predict the progression of several types of tumours [7] [8] [9], and this molecule has become the most widely adopted indicator of tumour progression [10].

PD-L1 is expressed not only on the surface of cells but also in soluble form in the circulation, with the amount in circulation being related to the number of PD-L1-expressing cells [11]. Similar to membranebinding PD-L1, soluble PD-L1 (sPD-L1) has a predictive value for cancer [12], with a high level of sPD-L1 being associated with an increased risk of tumour progression [13]. Circulating soluble PD-1 (sPD-1) in the bloodstream is believed to block the PD-1/PD-L1 signalling pathway and is thus used as an indicator of immune balance [14].

Accumulating studies have characterized the expression of PD-L1 in glioma [15] [16]. PD-L1 expression in tissue was found to be positively associ-ated with WHO grade [17] and described as a negative prognostic marker in glioma patients [18]. These findings raised our interest in determining whether soluble immune checkpoints can serve as biomarkers, as do their membrane-bound forms, in gliomas.

In the present study, we addressed three hypotheses through clinical comparisons and found the following: (a) sPD-1 and sPD-L1 levels in the circulation were higher in glioma patients than in healthy controls; (b) the levels were modified by surgical resection, and (c) sPD-1 and sPD-L1 in peripheral blood have the potential to predict glioma recurrence. This report provides insight into potential biomarkers for diagnosis and prognosis in gliomas.

## Materials and Methods

The study was approved by the Ethics Committee of Beijing Tiantan Hospital. Written informed consent for the procedures and publishing of data was obtained from the patients and their families. All procedures were performed after obtaining informed consent in accordance with the approved protocol.

### Patients and samples

This study enrolled two independent groups of glioma patients from Beijing Tiantan Hospital who received maximal safe resection and had histologically confirmed glioma.

Patients with brain tumours who underwent magnetic resonance imaging (MRI) between January 2016 and April 2018 were selected, and blood samples were prospectively collected before their surgical resections. Seventy patients with pathologically confirmed gliomas were incorporated into the pre-operative (Pre) group. Tumour volumes were measured via T2/FLAIR brain MRI.

The Pre group included six patients with gangliogliomas (GGs), 19 with low-grade astrocytomas (LGAs), 7 with oligodendrogliomas (ODGs), 12 with anaplastic gliomas (AGs) and 26 with GBMs. According to the WHO classification, 70 glioma patients were divided into grade I (8, 11.4%), grade II (23, 32.9%), grade III (13, 18.6%) and grade IV (26, 37.1%). Patients were followed-up to evaluate progression by brain MRI examinations. Progression was defined according to the Response Assessment in Neuro-Oncology (RANO) criteria, and progressionfree survival (PFS) was defined as the date of resection to the date of progressive disease or death [19]. PFS was the endpoint.

An independent cohort of 90 patients with GBM was used as the post-operative (Post) group. These patients were initially diagnosed between 2013 and 2017, and blood samples were obtained from each subject 5 to 15 days after surgery. The clinical data were extracted from medical records.

All cases were diagnosed histologically based on the 2007 WHO classification guidelines by the Department of Pathology at the Beijing Tiantan Hospital. Patients were eligible for this study if they had a newly diagnosed glioma and a complete surgical resection. None of the patients received chemotherapy, radiation or other immunotherapy.

We also analysed 20 healthy volunteers as a healthy control (HC) group; these volunteers had no cancer and no chronic diseases and were recruited during routine periodic medical examinations.

### Blood sampling

Patients' serum was obtained from residual blood samples that had been collected for other laboratory tests. To remove blood cells, serum tubes were centrifuged at 3,000 revolutions per minute for 10 min at room temperature. Separated serum samples were immediately stored at -80 °C until analysis. The repeated freezing and thawing of samples was avoided.

### Measurement of serum sPD-1 and sPD-L1

Serum levels of both sPD-1 and sPD-L1 were examined using specific enzyme-linked immunosorbent assay (ELISA) kits from Cusabio Biotech (Wuhan, China) according to the manufacturer's protocol. Each sample was tested in duplicate. The detection limit for ELISA was 3.9 pg/mL.

### Statistical analysis

The variables were investigated using Shapiro-Wilk tests to determine whether they were normally distributed. Continuous variables were displayed as the mean ± standard deviation (SD) or median with the minimum-maximum range. As most of the variables were not normally distributed, nonparametric methods were used for analysis. Differences in serum levels between cohorts were determined by using the Mann-Whitney or Kruskal-Wallis test. For the subanalysis of a statistically significant test, Bonferroni correction was used. Cut-off values for defining the high and low values of each variable were determined using receiver operating characteristic (ROC) analysis. PFS curves were performed using the Kaplan-Meier (K-M) method. A log-rank test was adopted to compare the survival curves. In the box plots, the horizontal lines indicate the median.

Statistical analyses were performed with SPSS (version 24.0, IBM, New York, USA). Statistical significance was defined as p<0.05 (*) or p<0.001 (***). Figures were constructed using GraphPad Prism (version 7, GraphPad Software, San Diego, California, USA).

## Results

### Patient characteristics

A total of 160 patients with glioma were included in the cohort study. In the Pre group, there were 70 patients representing cases of gliomas before resection. In the Post group, there were 90 glioblastoma patients whose blood samples were obtained 5-15 days after surgery. A total of 20 healthy volunteers were enrolled in the HC group. The characteristics and concentrations of sPD-1 and sPD-L1 are summarized in Table 1.

**Table 1:** Baseline patient characteristics and serum levels in different groups. Kruskal-Wallis test ^a^Median (min-max) ^b^Statistically significant

	Pre group	Post group	HC group	p
N	70	90	20	–
Age (year)	40.41±16.37	45.30±15.48	37.65±11.81	0.068
Sex (M/F)	39/31	57/33	10/10	0.432
Tumour size (cm^3^)	45.80 (1.50–472.50)	71.30 (4.00–490.00)	-	0.181
sPD-1 (pg/mL)^a^	11.28 (3.20–80.24)	8.79 (0–63.13)	6.42 (0–31.31)	<0.001^b^
sPD-L1 (pg/mL)^a^	49.96 (22.08–82.13)	8.97 (0–55.98)	34.48 (23.00–45.64)	<0.001^b^

**Figure 1:**
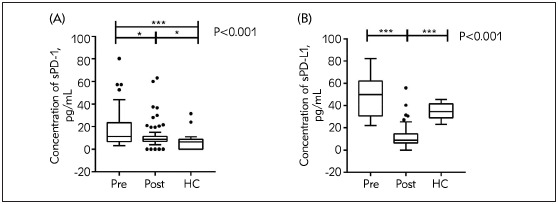
Serum levels of markers in glioma patients and healthy controls. (A) Serum sPD-1. (B) Serum sPD-L1.

As shown in Figure 1, no difference was found in age or sex among the three groups. The intergroup differences in the concentration medians of both sPD-1 and sPD-L1 were statistically significant (p<0.001). Additionally, we found more significant levels of the soluble proteins in the serum of patients who received resections than in those who did not (Figure 1).

Serum sPD-1 levels were significantly higher in the Pre group than in the HC group (p<0.001) and Post group (p=0.050). Compared with the HC group, the Post group had higher median sPD-1 levels (p=0.039) (Figure 1A).

The sPD-L1 levels in the Post group were significantly lower than those in the Pre group (p<0.001) and HC group (p<0.001). The sPD-L1 concentration did not differ significantly between the Pre and HC groups (p=0.369) (Figure 1B).

### The diagnostic value of sPD-1 and sPD-L1 in glioma

The ROC curve and area under the curve (AUC) were used to assess the ability of the soluble molecules to allow discrimination between patients and healthy subjects.

**Figure 2:**
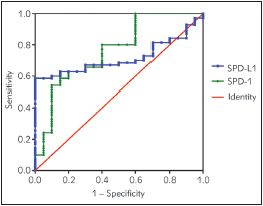
ROC curve analysis of serum sPD-1 and sPD-L1 concentrations in newly diagnosed glioma patients.

The optimal cut-off value of sPD-1 was 11.05 pg/mL, as defined by ROC curves for newly diagnosed glioma patients, with an AUC value of 0.762 (95% confidence interval (CI) 0.638-0.886, p<0.001). The optimal cut-off value for sPD-L1 was 46.16 pg/mL, with an AUC of 0.718 (95% CI 0.616-0.819, p=0.003) (Figure 2).

### Correlations between serum concentrations and disease features

Clinicopathological information of all 70 patients in the Pre group was recorded. Comparisons of serum sPD-1 and sPD-L1 levels in each disease feature are shown in Table 2. There were no significant differences in the levels of either soluble marker concerning sex, age, and tumour size (all p>0.05).

**Table 2:** Correlations between serum concentrations and disease features. Mann-Whitney U test & Kruskal-Wallis test ^a^ Median (min-max) ^b^ Statistically significant

Variable	Category	Cases	sPD-1 (pg/mL)^a^	p value	sPD-L1 (pg/mL)^a^	p value
Age (years)	≤18	11	7.28 (5.27–30.11)	0.556	50.06 (24.88–71.92)	0.898
	19–60	47	11.36 (3.20–57.24)		49.57 (22.08–82.13)	
	>60	12	23.55 (5.24–80.24)		53.98 (23.24–80.82)	
Sex	Male	39	11.36 (3.36–52.64)	0.718	52.32 (22.08–82.13)	0.732
	female	31	11.20 (3.20–80.24)		49.47 (22.53–80.82)	
Tumour size (cm^3^)	45.8	35	10.51 (3.20–80.24)	0.545	45.12 (22.08–82.13)	0.681
	≥45.8	35	14.13 (4.92– 44.08)		53.96 (22.53–79.53)	
Grade (WHO)	I	8	6.18 (3.36 –15.21)	0.000^b^	31.12 (25.28–59.63)	0.027^b^
	II	23	7.68 (3.20–52.64)		43.98 (22.53–73.34)	
	III	13	11.36 (4.78–57.24)		49.57 (23.62–71.45)	
	IV	26	23.58 (6.82–80.24)		59.58 (22.08–82.13)	
Histopathological type	GG	6	7.34 (4.20 –15.21)	0.000^b^	31.12 (25.28–59.63)	0.057
	LGA	19	6.89 (3.20–57.24)		40.31 (22.53–73.34)	
	ODG	7	8.92 (4.92–14.13)		56.43 (23.08–65.76)	
	OG	12	10.94 (4.78–28.72)		47.34 (23.62–62.80)	
	GBM	26	23.58 (6.82–80.24)		57.58 (22.08–82.13)	
Recurrence	(+)	21	23.20 (6.89–44.08)	0.000^b^	54.00 (22.08–82.13)	0.198
	(–)	49	8.34 (3.20–80.24)		49.57 (22.53–80.82)	

As shown in Table 2, the concentrations of sPD-1 and sPD-L1 differed significantly according to grade (mild vs advanced) (p<0.001 and p=0.027, respectively). In addition, sPD-1 levels differed among histopathological types (p<0.001). Although sPD-L1 level varied by pathological type, no significant difference was observed (p=0.057).

**Figure 3:**
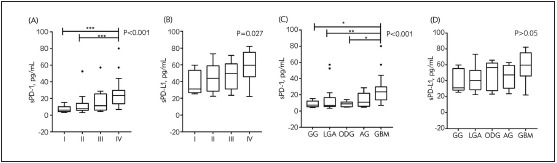
The diversity of serum levels in different glioma grades and different morphological types. (A & C) Soluble PD-1. (B & D) Soluble PD-L1.

As indicated in Figure 3, the concentration of sPD-1 was increased in GBM (WHO grade IV) relative to low-grade gliomas, such as ODG (grade I), LGA and ODG (grade II). Interestingly, sPD-L1 level was much higher in GBM than in the other lower-grade gliomas, although the differences were not significant.

### Prognostic potential of sPD-1/sPD-L1 for postsurgery glioma recurrence

At a median follow-up time of 17.0 months (95% CI 17.66-21.0), postoperative recurrence had occurred in 21 patients at the time of data analysis.

**Figure 4:**
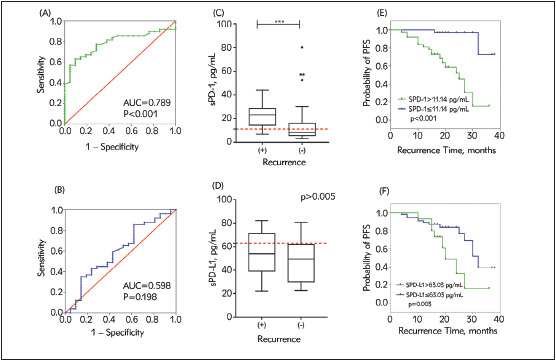
(A & B) ROC curve analyses of serum sPD-1 and sPD-L1 levels for predicting glioma recurrence. (C & D) Serum levels of each marker in patients with recurrence and progression-free survivors; the red dashed lines indicate the optimal cut-off values. (E & F) Kaplan-Meier analysis of PFS in patients with high and low serum levels of sPD-1 and sPD-L1.

As shown in Table 2, a significant difference was observed in sPD-1 level between glioma-recurrence patients and progression-free survivors. Differences in sPD-L1 levels were not observed among the pathological types. In Figure 4, box plots of each soluble molecule are presented to the right of their corresponding ROC curves (Figure 4C & Figure 4D).

We employed ROC curve analysis to assess the ability of serum sPD-1 and sPD-L1 to predict recurrence. As shown in Figure 3A & Figure 3B, the optimal cut-off value of sPD-1 was 11.14 pg/mL, as defined by ROC curves for tumour recurrence, with an AUC value of 0.789 (95% CI 0.683-0.894, p<0.001). The optimal cut-off value for sPD-L1 was 63.03 pg/mL, with an AUC value of 0.598 (95% CI 0.448-0.748, p=0.198).

The patients were classified as having low or high levels of each marker by using the optimal thresholds mentioned above. For both sPD-1 and sPD-L1, we noted markedly significant differences in PFS between the high-and low-level groups (Figure 4E & Figure 4F)

Kaplan-Meyer analyses indicated that high sPD-1 concentrations (>11.14 pg/mL) predict shorter PFS times, with a median of 25.0 months for glioma patients (log-rank p<0.001) (Figure 4E).

Similarly, patients with a high level of sPD-L1 (>63.03 pg/mL) have a poorer PFS rate than patients with a low level of sPD-L1 (median progression time with high vs. low: 20.0 months vs 32.0 months; log-rank p = 0.030) (Figure 4F).

## Discussion

With the remarkable clinical success of immune checkpoint inhibitors in several refractory tumours, PD-1 and its ligand PD-L1 have generated increasing interest in exploring potential treatment targets and promising biomarkers in glioma therapies [20] [21]. In the present study, we characterized the expression of soluble PD-1 and PD-L1 in the serum of newly diagnosed glioma patients and investigated the potential predictive roles of these markers.

Prior studies indicated that the secretion of sPD-1 and sPD-L1 in sera might be relevant to the corresponding membrane-bound molecules on tumour cells or immune cells [22]. Therefore, we hypothesized that higher levels of these soluble molecules might be detected in the blood of tumour patients than in the blood of healthy individuals. As expected, elevated sPD-1 and sPD-L1 levels were detected in the pre-operative sera of 70 glioma patients relative to the levels in healthy controls. In addition, we observed markedly decreased circulating levels in 90 GBM patients after tumour resections. The preliminary results suggested that the decrease in concentrations might be related to the removal of the tumour. We performed a ROC curve analysis to identify the optimal cut-off value for glioma diagnosis. The AUC of sPD-1 level was considered adequate for diagnosing gliomas and was similar to that of sPD-L1 (0.762 and 0.718, respectively). These results support sPD-1 and sPD-L1 as potential diagnostic markers.

Recent studies have shown that PD-1 and PD-L1 are highly expressed in malignant gliomas and that their expression is closely associated with malignant grades and adverse outcomes [23] [24]. Consistent with these studies, our data revealed trends of higher serum sPD-1 and sPD-L1 levels with increasing malignancy grade. Notably, we found that patients with GBM showed markedly higher serum levels of sPD-1/sPD-L1 than did patients with lowergrade gliomas. Some studies have shown that high levels of soluble PD-1 and PD-L1 are associated with worse clinical outcomes in several types of tumours [25] [26]; moreover, researchers have speculated that the expression of these markers in peripheral blood reflects the immune environment and have shown them to be highly correlated with malignant neoplasm behaviour [7]. Since gliomas are the most aggressive form of brain neoplasms, with a high recurrence rate, we speculate that high serum levels of sPD-1/PD-L1 in gliomas might have the potential to predict tumour recurrence and progression.

ROC curves were constructed to assess the efficacy of sPD-1 and sPD-L1 in predicting glioma recurrence. Although sPD-L1 failed to predict tumour recurrence (p>0.05), K-M analysis demonstrated that higher levels of sPD-L1 (higher than the threshold level determined from the ROC curves) were correlated with shorter PFS. Additionally, serum sPD-1 levels demonstrated good prognostic performance for PFS as revealed by ROC curve and K-M analyses. Therefore, circulating immune checkpoint parameters might serve as alternative markers for monitoring tumour aggressiveness in the post-surgery management of glioma.

However, the differences in circulating sPD-L1 level among histologic types in our research were not significant (p>0.05). In addition, we found that tumour size was not parallel to the serum level of sPD-1 or sPD-L1 before resection, potentially due to the protective blood barrier; thus, the concentrations of these molecules in sera do not reflect tumour size. Another potential reason for the lack of observed differences in circulating sPD-L1 level among histologic types is that sPD-1 and sPD-L1 are not exclusive to tumour cells [27] and are released by diverse cell populations through different mechanisms, including intrinsic splicing [13] [28] and the cleavage of membrane-bound counterparts [11].

Although our study detected two potential markers for diagnosing and predicting recurrence in glioma patients, there are some limitations to the study. First, the composition of grades and the small number of patients limited our ability to detect and describe the predictive power of these soluble markers. Second, the clinical data of glioma patients lacked molecular classification in our hospital. As molecular classification paves the way for more precise medicine for gliomas [29], further research should attempt to investigate the associations of molecular markers with circulating sPD-1/PD-L1 levels in the peripheral blood of glioma patients.

## Conclusions

This study found elevated levels of sPD-1 and sPD-L1 in the serum of glioma patients relative to the levels in healthy patients, and these levels might be modified by tumour removal. In addition, higher serum sPD-1/sPD-L1 levels showed a predictive capacity for diagnosis and progression. This study provides clinical data that can inform future studies of the potential of soluble PD-1 and PD-L1 as biomarkers in malignant brain tumours.

*Acknowledgements*. This work was supported by the National Science and Technology Major Project of China (2018ZX10307415-003).

## Conflict of interest statement

The authors declare that they have no conflict of interest.
